# Assessment of allelic diversity in intron-containing Mal d 1 genes and their association to apple allergenicity

**DOI:** 10.1186/1471-2229-8-116

**Published:** 2008-11-13

**Authors:** Zhongshan Gao, Eric W van de Weg, Catarina I Matos, Paul Arens, Suzanne THP Bolhaar, Andre C Knulst, Yinghui Li, Karin Hoffmann-Sommergruber, Luud JWJ Gilissen

**Affiliations:** 1 Department of Horticulture/Allergy Research Center, Zhejiang University, Hangzhou 310029, PR China; 2 Allergy Consortium Wageningen, Wageningen University and Research Centre, P.O. Box 16, 6700AA, Wageningen, the Netherlands; 3 Plant Research International, Wageningen University and Research Centre, P.O. Box 16, 6700AA, Wageningen, the Netherlands; 4 Department of Dermatology/Allergology, University Medical Center Utrecht, P.O. Box 85500, 3508GA Utrecht, the Netherlands; 5 The National Key Facility for Crop Gene Resources and Genetic Improvement (NFCRI)/Key Lab of Germplasm & Biotechnology (MOA), Institute of Crop Science, China Academy of Agricultural Science, Beijing, 100081, PR China; 6 Department of Pathophysiology, Medical University of Vienna, AKH-EBO-3Q, Währinger Gürtel 18-20, A-1090 Vienna, Austria

## Abstract

**Background:**

Mal d 1 is a major apple allergen causing food allergic symptoms of the oral allergy syndrome (OAS) in birch-pollen sensitised patients. The *Mal d 1 *gene family is known to have at least 7 intron-containing and 11 intronless members that have been mapped in clusters on three linkage groups. In this study, the allelic diversity of the seven intron-containing *Mal d 1 *genes was assessed among a set of apple cultivars by sequencing or indirectly through pedigree genotyping. Protein variant constitutions were subsequently compared with **S**kin **P**rick **T**est (SPT) responses to study the association of deduced protein variants with allergenicity in a set of 14 cultivars.

**Results:**

From the seven intron-containing *Mal d 1 *genes investigated, *Mal d 1.01 *and *Mal d 1.02 *were highly conserved, as nine out of ten cultivars coded for the same protein variant, while only one cultivar coded for a second variant. *Mal d 1.04*, *Mal d 1.05 *and *Mal d 1.06 A, B *and *C *were more variable, coding for three to six different protein variants. Comparison of *Mal d 1 *allelic composition between the high-allergenic cultivar Golden Delicious and the low-allergenic cultivars Santana and Priscilla, which are linked in pedigree, showed an association between the protein variants coded by the *Mal d 1.04 *and *-1.06A *genes (both located on linkage group 16) with allergenicity. This association was confirmed in 10 other cultivars. In addition, *Mal d 1.06A *allele dosage effects associated with the degree of allergenicity based on prick to prick testing. Conversely, no associations were observed for the protein variants coded by the *Mal d 1.01 *(on linkage group 13), -*1.02*, -*1.06B, -1.06C *genes (all on linkage group 16), nor by the *Mal d 1.05 *gene (on linkage group 6).

**Conclusion:**

Protein variant compositions of Mal d 1.04 and -1.06A and, in case of *Mal d 1.06A*, allele doses are associated with the differences in allergenicity among fourteen apple cultivars. This information indicates the involvement of qualitative as well as quantitative factors in allergenicity and warrants further research in the relative importance of quantitative and qualitative aspects of *Mal d 1 *gene expression on allergenicity. Results from this study have implications for medical diagnostics, immunotherapy, clinical research and breeding schemes for new hypo-allergenic cultivars.

## Background

Many birch pollen sensitised patients (50–70%) in Central and Northern Europe suffer from oral allergy symptoms after eating fresh apples [1]. The prevalence of apple allergic individuals mounts up to ~3% in Central and Northern Europe. This type of apple allergy is caused by cross reactivity of IgE antibodies against the major and sensitizing birch pollen allergen Bet v 1 with Mal d 1, the major allergen of apple. Bet v 1 and Mal d 1 are both pathogenesis-related (PR) proteins. They belong to the PR-10 family and share a high degree of homology [2-6].

From patients' experience it is known for a long time that the severity of allergic reactions to apple was not only related to the specific sensitivity of the individual, but also largely depended on the apple cultivar. This cultivar dependent allergenicity has also been described in literature. For instance, Mal d 1 from the cultivar Golden Delicious was found highly reactive to specific IgE antibodies from allergic patients' sera, whereas Mal d 1 from the cultivar Gloster generally showed much less reactivity [7,8]. In addition, skin prick testing (SPT) with 21 different apple cultivars and confirmations for specific cultivars in double-blind placebo-controlled food challenges (DBPCFC) and oral challenges of whole apples, revealed a wide range of allergenic reactivity from very high to very low [9,10]. As a result from these studies, the new cultivar Santana was identified as hypo- allergenic for 75% of the patients with a mild apple allergy [10], which is usually assumed to be Mal d 1 based. In The Netherlands (where birch pollen-related apple allergy is by far the most common form of apple allergy), this cultivar has recently been marketed as 'suited for individuals with mild apple allergy' in order to meet the general desire of apple allergic persons to be able to add this common fruit to their daily diet.

The differences in allergenicity among cultivars raised a crucial question on the origin of this cultivar-specific degree of allergenicity. Allergenicity may depend on the total amount of Mal d 1 proteins, as suggested by Son et al. [11] from their observed ten-fold difference in Mal d 1 amount between the high-allergenic cultivar Golden Delicious and the low-allergenic cultivar Gloster. However, there is little evidence supporting this hypothesis because only very few cultivars have been studied and, for these, a linear response between Mal d 1 protein content and allergenicity estimates is lacking. On the other hand, qualitative characteristics of the Mal d 1 proteins could be involved too, as can be argued from the differences in binding capacity of birch pollen-specific IgE to two protein variants of Mal d 1 [11-13]. To elaborate this latter issue, research on the genetic variation of Mal d 1 and its expression pattern in the different cultivars is required and should be compared to allergenicity data. It is known that Mal d 1 is coded by a large gene family of 18 members mapped on three linkage groups of the apple genome [14,15]. Not all of these members are likely to be involved in allergenicity since only a limited number of different Mal d 1 proteins and mRNAs have been traced back in apple fruit so far [16-18].

Research towards the relative importance of the quality and quantity of Mal d 1 proteins on the allergenicity of apple cultivars is relevant for designing apple breeding programs for low-allergenic apple cultivars of high quality and healthiness. In this paper, we focused on the genetic diversity of *Mal d 1 *genes. The *Mal d 1 *gene family can be subdivided into two major categories: genes with and genes without an intron. Preliminary genetic analyses revealed that the genetic diversity was by far larger in the intron-containing genes. Furthermore, the intron-containing genes cover all three linkage groups that *Mal d 1 *loci [15]. Therefore, this category of genes has been chosen to start looking for putative qualitative effects of Mal d 1 proteins in cultivar specific allergenicity.

Allelic diversity of the seven intron-containing *Mal d 1 *genes was assessed among a set of cultivars chosen for their importance in breeding programs and apple production. In order to find putative associations with allergenicity, the presence of alleles coding for different protein variants was subsequently compared with the degree of allergenicity for a subset of cultivars for which allergenicity data from SPT or DBPCFC tests were available.

## Results

### Diversity of *Mal d 1 *genes and deduced proteins

The observed DNA polymorphisms in the 10 studied cultivars resulted in a total of 46 different *Mal d 1 *sequences over seven genes (Table 1, 2). These sequences were denoted according to the occurrence of 1) DNA polymorphisms in the coding region of the gene leading to different protein variants; 2) polymorphism in the coding region that did not affect the protein sequence (silent mutations), and 3) polymorphism in the intron (Table 1, 2). Although the latter two differences are of minor importance with respect to allergenicity, they provided additional landmarks for the development of sequence specific molecular markers.

**Table 1 T1:** Genetic variation in the intron containing *Mal d 1 *genes of linkage groups 6 (*Mal d 1.05*) and 13 (*Mal d 1.01*) among ten apple cultivars.

**Iso-allergen**	**Variant^a^**	**Sil. mut**	**gDNA #^b^**	**Cultivar^c^**										**GenBank Accessions**
					
				**GD**	**PS**	**IM**	**CO**	**JO**	**RD**	**FJ**	**DS**	**PM**	**FS**	
Mal d 1.05	01	1	1	+^d^	+	+ +	+ +	+	+		+	+ +	+	AY789245–AY789246, AY827676–AY827682
	02	1	2								+		+	AY789247, AY827683
	02	2	3	+	+									AY827684–AY827685
	03	1	4					+	+	+ +				AY827686–AY827688

Mal d 1.01	05	1	1									+		AY789236
	05	1	2	+				+		+				AY827639–AY827641
	05	2	3	+	+	+ +	+ +	+			+ +		+ +	AY789238, AY827633–AY827638
	05	3	4		+							+		AY789237, AY827642
	05	4	5						+	+				AY827643–AY827644
	09	1	6						+					AY827645

^a^Variants refer to different protein sequences. Variant numbers of Mal d 1.01, Mal d 1.02 and Mal d 1.04 are according to the allergen list (; Sept 2008), those for Mal d 1.05 and Mal d 1.06 are according to Gao et al. [15].

^b^Genome sequences are numbered successively per gene

^c^Golden Delicious (GD), Priscilla (PS), Ingrid Marie (IM), Cox (CO), Jonathan (JO), Red Delicious (RD), Fuji (FJ), Discovery (DS), Prima(PM) and Fiesta (FS). Note that different accessions of Priscilla seem to exist. The Priscilla used here is a parent of Santana as confirmed by 20 SSR markers (unpublished data), but can not descend from its supposed mother Starking Delicious

^d ^Indicates the presence of an allele in heterozygous (+) or homozygous (+ +) condition.

**Table 2:** Genetic variation in the intron containing *Mal d 1 *genes of linkage group 16 among ten apple cultivars.

**Iso-allergen**	**Variant^a^**	**Sil. mut**	**gDNA #^b^**	**Cultivar^c^**										**GenBank Accns**
					
				**GD**	**PS**	**IM**	**CO**	**JO**	**RD**	**FJ**	**DS**	**PM**	**FS**	
1.02	01	1	1	#			#	#				#	# *	AY789240–AY789241, AY827646–AY827648
	01	2	2									*		AY789239
	01	3	3		# *	*	*	*	*	*				AY827649–AY827654
	01	4	4	*						#				AY827655–AY827656
	01	5	5			#								AY827657
	01	6	6						#					AY827658
	01	7	7								+			AY827659
	09	1	8								+			AY827660

1.04	04	1	1									*		AY789242
	04	2	2	# ^e^			#	#	#			#	# *	AY789243–AY789244, AY827661–AY827664
	05	1	3								+			AY827665
	06	1	4			#								AY827666
	07	1	5								+			AY827667
	ps1	1	6		# *			*	*	*				AY827668–AY827671
	ps2	1	7	*		*	*			#				AY827672–AY827675

1.06A	01	1	1–10	#			#	#					# *	AY789249, AY827689–AY827691
	01	1	2–11									#		AY789250
	02	1	3–16									*		AY789248
	02	2	4–6		# *	*	*	*	*	*				AY827692–AY827697
	02	3	5–10			#			#					AY827698–AY827699
	02	4	6–7								+			AY827700
	03	1	7–7	*						#	+			AY827701–AY827703

1.06B	01	1	1	#	#		#	#					# *	AY789251, AY827704–AY827707
	02	1	2	*	*			*	*	# *		#		AY789252, AY827708–AY827712
	03	1	3						#			*		AY789253, AY827713
	03	2	4			#								AY827714
	04	1	5			*	*							AY827715–AY827716
	05	1	6								+			AY827717
	05	2	7								+			AY827718

1.06C	01	1	1	#	#		#	#					# *	AY789254, AY827719–AY827722
	02^g^	1	2									*		AY789255
	03^g^	1	3	*	*			*	*	# *	+	#		AY789256, AY827723–AY827725
	04	1	4			#					+			AY827726
	05	1	5						#					AY827727
	06	1	6			*	*							AY827728

^a^Variant numbers of are according to the allergen list (see Table 1), those for Mal d 1.05 and Mal d 1.06 are according to Gao et al. [15].

^b, c, d ^as in Table 1

^e ^# and * For LG16, alleles that have the same symbol are in coupling phase with each other. This information was obtained by their co-segregation patterns in mapping progenies (Prima × Fiesta and Jonathan × Prima [15], and over pedigrees (Fiesta = Cox × Idared [= Jonathan × Wagner Apfel]); Ingrid Marie = Cox × open pollinated and Fuji = Ralls Janet × Delicious; Red Delicious is a colour mutant of Delicious.

^f^*ps1 *and *ps2 *of *Mal d 1.04 *refer to pseudo-alleles.

^g ^The allele specific markers for these alleles were derived from Table 3 of Gao et al. 2005 [15] whereby we took into account that their marker for *Mal d 1.06C02 *actually amplifies *Mal d 1.06C03 *and visa versa, thus harmonizing an inconsistency between their tables 2 and 3 in the assignment of allele and marker names to the AY789255 and AY789255 sequences.

Mal d 1.01 and -1.02 showed to be highly conserved at the protein level. The related genes coded for only two variants each that both differed in just a single amino acid (pos. 135 V/A for Mal d 1.01, pos. 56 N/K for Mal d 1.02) and for which the second variant was found only once. The other genes were more variable, coding for three (*Mal d 1.05*) up to six variants (*Mal d 1.06C*). *Mal d 1.04 *showed to be special in that two (out of the three) sequences contained a stop codon in the coding region and were therefore regarded as pseudo alleles (*ps1 *and *ps2*). Interestingly, the pseudo alleles occurred frequently as for seven out of the ten cultivars at least one of the alleles was a pseudo allele whereas cultivars Priscilla and Fuji only contained pseudo alleles of *Mal d 1.04 *(Table 2).

### Allergenicity scores of 14 apple cultivars by skin prick test (SPT)

Relative SPT responses of 14 apple cultivars are given in Table 3. Fiesta, Delblush, Pinova and Golden Delicious were ranked in the high allergenic group (83–100%). Priscilla and Santana showed low SPT responses, with wheal areas 30–35% of that of Golden Delicious. Nine cultivars were intermediate (48–72%) allergenic. Santana was also identified as low-allergenic in comparison to Golden Delicious in DBPCFC tests [9] and oral provocation tests [10].

**Table 3:** SPT responses of apple cultivars relative to Golden Delicious (in %) for 4 experiments.

Cultivar	Experiment^a^				Average
		
	I	II	III	IV	
Priscilla				30	30
Santana	34		38	30	34
Jonathan				48	48
Ecolette	39	62			51
Prima				61	61
Elstar	67		52		60
Fuji	70	69	48		62
Gala	65		62		63
Elize Roblos	67				67
Bellida	72				72
Fiesta	67			99	83
Delblush Tentation	87				87
Pinova	89				89
Golden Delicious	100^b^	100	100	100	100

^a ^Data for the first three experiments were derived from the original data from three experiments of Bolhaar et al. [9] on respectively cultivar screening, intra-cultivar variation and storage, of which we used only the data of patients with mild symptoms. The forth experiment came from a new cultivar screening experiment.

^b ^The underlying HEP values for Golden Delicious for the four experiments were respectively 0.54, 0.45, 0.63 and 0.69.

### General associations from the sequenced cultivars

From the cultivars used to sequence the intron containing *Mal d 1 *genes and to perform SPT on allergenicity, Golden Delicious was ranked as the highest allergenic cultivar whereas Priscilla was ranked as the lowest (Table 3). This difference was not related to protein variant composition of Mal d 1.01, 1.05, 106B and 1.06C because these cultivars have identical protein variants (Table 2). In contrast, both Mal d 1.04 and Mal d 1.06A have different protein variants in Golden Delicious and Priscilla. Mal d 1.04 may contribute to the observed difference in allergenicity between the two cultivars since protein variant 04 is present in Golden Delicious while Priscilla is heterozygous for the two pseudo-alleles *ps1 *and *ps2*. Similarly, Mal d 1.06A protein variant -02 was found in Priscilla whereas variants -01 and -03 were found in Golden Delicious. The intermediate cultivars, Jonathan, Prima, and Fuji, have the putatively high allergenic alleles *-01 *or -*03 *in single dosage, whereas high allergenic cultivars either have both alleles *-01 *and *-03*, or have *-01 *in homozygous state. This suggests that allergenicity might be determined by both the protein variant and the gene dosage of highly allergenic alleles.

### Association analysis by pedigrees: from Golden Delicious to Santana

**Figure 1:**
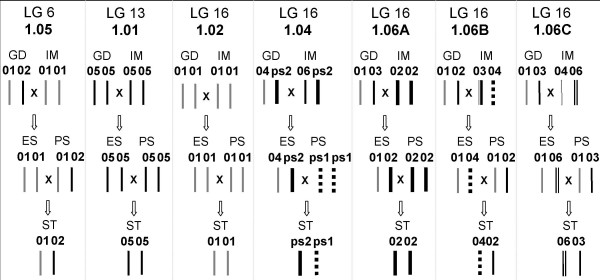
**Flow of putative protein variants over the pedigree of Santana for seven *Mal d 1 *genes**. Abbreviated cultivar names are according to Table 1. GD is high allergenic whereas PS and ST are low allergenic [9,10].

The identity and origin of genomic alleles and thus protein variants in additional cultivars (not sequenced for *Mal d 1*) could be traced by developed allele specific SNAP and SSR markers and the use of pedigree information [19] (see Methods). For instance, the deduced flow of protein variants over the pedigree of cultivar Santana is presented in Figure 1. Santana and Priscilla are low allergenic whereas Golden Delicious is high allergenic [9,10]. For Mal d 1.01, 1.02 and 1.05 the same protein variants were found for Golden Delicious and Santana. In contrast, Golden Delicious and Santana differ in their protein variant composition of Mal d.1.04, -1.06A and -1.06B (Fig. 1), indicating a possible involvement of these proteins in the observed difference in allergenicity between these cultivars. Santana, like Priscilla, has only pseudo alleles for *Mal d 1.04 *that do not result in protein production, while Golden Delicious has one functional allele. The Mal d 1.06A02 variant of Santana is not present in Golden Delicious.

### Associations for Mal d 1.04 and -1.06A

Similarly to Santana, pedigree information and sequence specific markers were used to assess the putative protein variant composition for other additional cultivars for which we have SPT response data. This allowed a further validation of the association of the Mal d 1.04 and 1.06A with allergenicity in a total of 14 cultivars (Table 4). Cultivars Delblush and Pinova evoked an SPT response that was similar to that of Golden Delicious and also had the same variant compositions as Golden Delicious for Mal d 1.04 and Mal d 1.06A, namely variant 04 for Mal d 1.04 (coded for by a single allele) and the two variants 01 and 03 for Mal d 1.06. The remaining five cultivars evoking an intermediate allergenic response also had a similar variant composition as the previously identified intermediate cultivars, namely variants 02 and 03 for Mal d 1.06A, and variant 04 of Mal d 1.04 (coded for by a single allele dosage), except for Prima, that has two alleles coding for Mal d 1.0404 and Fuji that contains both pseudo alleles. The other intron containing genes did not show any association (data not shown).

**Table 4:** SPT responses of 14 apple cultivars and the putative protein constitutions of these cultivars for two Mal d 1 iso-allergens.

**Cultivar^a^**	**SPT-response^b^**	**Mal d 1.04**		**Mal d 1.06A**	
**Priscilla**	30	**ps1**	**ps2**	**02**	**02**
Santana	35	**ps1**	**ps1**	**02**	**02**
**Jonathan**	48	**ps1**	04	01	**02**
Ecolette	51	**ps2**	04	01	**02**
**Prima**	61	04	04	01	**02**
Elstar	61	**ps2**	04	01	**02**
**Fuji**	61	**ps1**	**ps2**	03	**02**
Gala	64	**ps1/ps2**	04	01	**02**
Elise	67	**ps2**	04	01	**02**
Bellida	72	04	04	01	01
**Fiesta**	83	**ps1**	04^3^	01	01
Delblush	87	**ps2**	04	01	03
Pinova	89	04	**ps2**	01	*03*
**Golden Delicious**	100	**ps2**	04	01	*03*

^a^: Cultivars in bold were sequenced for their intron containing *Mal d 1 *genes

^b^: In % relative to Golden Delicious.

## Discussion

Birch pollen induced oral allergy for apple has been the subject in a considerable number of studies. One of the prominent results has been the presence of cultivar-specific differences in allergenicity. Unfortunately, evidence regarding the causes of cultivar-specific allergenicity is still lacking. One of the knowledge gaps concerned the number and identity of *Mal d 1 *genes and the amount of variation within these genes. Recently, Gao et al. [15] have shown that *Mal d 1 *genes are members of a large gene family by identifying 18 different loci that are located in three clusters. Based on sequence identity, these 18 genes could be subdivided into intron containing and intronless genes. In order to create a basis for a better understanding of the genetics of *Mal d 1 *genes and their impact on allergenicity, we have studied the allelic diversity of the intron containing genes in 10 cultivars that are often used in breeding. Development of sequence specific markers and pedigree information enabled the assessment of putative *Mal d 1 *constitutions of other cultivars. Using this information, we assessed the different Mal d 1 isoforms that cultivars are able to produce and found associations between their putative protein constitutions and SPT-responses.

### Allelic diversity and validity of database sequences

Cloning and sequencing of the seven intron-containing *Mal d 1 *genes in 10 cultivars revealed 46 different alleles that coded for 25 different Mal d 1 isoforms. The variation per gene varied with regard to the number of alleles and deduced proteins. *Mal d 1.01 *and *Mal d 1.02 *were diverse at the gDNA level but conserved at the protein level. For both genes only two protein variants were found, of which the second variant, differing in one amino acid only, was found in just a single cultivar. For *Mal d 1.04*, *Mal d 1.05 *and the three *Mal d 1.06 *genes, gDNAs often coded for different proteins and these genes were therefore more variable at the protein level.

Because the examined cultivars are important in the breeding of many modern apple varieties, the set of alleles found in this study likely represents a considerable part of the total variation present in intron containing *Mal d 1 *genes of common apple varieties.

Although other *Mal d 1 *sequences are known from public databases, we suspect that many of these sequences may be artefacts derived through strand switching and PCR mutations. The problem associated with PCR amplification of a group of closely related sequences, such as the *Mal d 1 *gene family, is that besides PCR induced single base pair mutations, in vitro strand switching or re-annealing of incompletely amplified fragments can lead to artefacts as was exemplified by Schenk et al. [20] for birch Bet v1 sequences. For instance, for *Mal d 1.01 *one of the most studied *Mal d 1 *genes, over 13 DNA sequences from previous studies are known from public databases (Table 5) indicating the presence of 9 putative protein isoforms. We know *Mal d 1.01 *is a single locus gene with maximum two alleles present in a cultivar [15], but four sequences from Golden Delicious can be found in databases. From these, only sequence accession AF124830 was identical to one of our two sequences (Mal d 1.0105.01b). The other sequences may be due to artefacts. Firstly, accession AF126402 had one SNP at position 11 (G→A) compared to AF124830, which is due to the cloning primer used. Similarly, sequences from a number of other cultivars showed this 11A mutation too. In our study, the cloning primers used were positioned in the 5'-untranslated region thus avoiding this problem. Secondly, Accession Z48969[21] that was classified as Mal d 1c [10] is quite similar to *Mal d 1.01*, while the SNPs found are unique to three other *Mal d 1 *genes. This may be due to an artefact from strand switching during PCR which could explain why we, like other researchers could not retrieve this sequence from Golden Delicious [[10,17]; this study]. Similarly, the first 84 nucleotides of AF124829 and AF124832 match to *Mal d 1.02 *(typical SNPs are shown at the bottom of Table 5), while the remaining sequence matches to *Mal d 1.01 *making these accessions also putative erroneous sequences due to strand switching. Therefore, the actual number of *Mal d 1.01 *variants present among apple cultivars is probably not as high as nine, because at least five of them are likely PCR artefacts. Two protein variants (1.0105 and 1.0109) of *Mal d 1.01 *could be confirmed in this study. Variants Mal d 1.0104, 1.0107 and 1.0108 are likely artefacts of 1.0105 whereas 1.0101 and 1.0102 are likely artefacts of 1.0109.

**Table 5:** SNPs among *Mal d 1.01 *sequences as found in databases.

Allele	Cultivar^b^	Nucleotide position in coding sequences^c^																							
		
(Genbank accession no)^a^		1 1	2 1	2 9	4 9	7 5	8 4	2 2 2	2 9 4	3 3 4	3 6 0	3 6 4	4 0 4	4 0 5	4 0 8	4 1 3	4 1 9	4 2 0	4 3 5	4 5 3	4 5 6	4 5 8	4 6 5	4 6 8	4 7 1
		Consensus nucleotide																							
		G	G	T	T	T	C	G	A	T	C	A	C	T	A	A	C	C	G	T	G	A	C	C	A
Mal d 1.0101 (X83672)	GS	*A*											T												
Mal d 1.0102 (Z48969)	GD	*A*	*A*							*G*		*C*	T	*C*			*G*	*T*		*C*		*G*	*G*	*T*	*C*
Mal d 1.0103 (AF124823)	JB	*A*						A											A		*A*	*G*			
Mal d 1.0104 (AF124829)	JG	*A*		*A*	*C*	*C*	*T*				T														
**Mal d 1.0105.01 **(AF124830, AY428579)	GD, GA, **PM, GD, JO, FJ**										**T**														
**Mal d 1.0105.02**	**PM, FS, GD, PS, IM, JO, CO, DS**							**A**											**A**						
**Mal d 1.0105.03**	**PM, PS**																								
**Mal d 1.0105.04**	**RD, FJ**							**A**																	
Mal d 1.0106 (AF124831)	GL								C																
Mal d 1.0107 (AF124832)	GA	*A*		*A*		*C*	*T*	A							*C*	*C*		*T*		*C*					
Mal d 1.0108 (AF126402)	GD	*A*									T														
Mal d 1.0109 (AY026910)	GD-seedling, **RD**												**T**												

**Mal d 1.02-CONS^d^**		A	G	A	C	C	T	G	G	TC	C	A	C	T	A	A	C	T	G	C/T	G	G	C	C	A

^a ^Alleles in bold are confirmed by our own sequences (see Table [Table T1]). Numbers in brackets indicate Genbank accessions of previous sequences.

^b ^Cultivar abbreviations additional to those in Table 2: GA-Gala, JB-Jamba, GL-Gloster, GD-seedling: seedling from Golden Delicious. Cultivar names in bold indicate material from this study.

^c^Position refers to the coding sequence and is presented vertically. SNP nucleotides given in bold are our own observations. SNPs in italic are identified errors after cross checking.

^d^*Mal d 1.02 *consensus SNPs compared with *Mal d 1.01*.

The occurrence of PCR recombination and mutations in sequences from gene families warrants scrutinised assessment of sequences. The use of two independent PCR-cloning steps for each cultivar may effectively filter out most of these erroneous sequences before database donation since the probability of isolating identical artefacts in independent PCRs is low [20]. Sequence specific markers may be used to validate newly found isoforms that have passed this first sifting. Many of the sequences found in this study were either confirmed by identical sequences retrieved from the other cultivars used or by identical sequences previously donated in the databases as well as through the use of sequence specific markers [[15], this study].

### Cultivar specific allergenicity and its relation to quantitative and qualitative differences in Mal d 1

Levels of total Mal d 1 protein can not fully explain allergenicity of cultivars, indicating that other factors are involved as well. For instance, Son et al. [10] found a fourfold higher level of Mal d 1 protein in Granny Smith compared to Gloster. Although this suggests a correlation between total amount and allergenicity, this relation was contradicted by their results on Golden Delicious and Granny Smith, which cultivars were similar allergenic despite of a threefold difference in total Mal d 1 content. Furthermore, it was demonstrated that different Mal d 1 isoforms [10], as well as mutants of specific isoforms [13], have different binding affinities to IgE indicating that the relative Mal d 1 protein composition as well as the presence of specific alleles may have a significant effect on allergenicity.

This research is the first to show an association between the genetic *Mal d 1 *constitution of apple cultivars and birch pollen related allergenicity. If the allergenicity is a matter of the variants of a single gene, then *Mal d 1.06A *will be the most outstanding candidate. In case of the involvement of multiple genes, also *Mal d 1.04 *may play a role. A putative involvement of the intronless genes can not yet be excluded, as their allelic variation has not yet been surveyed. Such a survey will be a challenge considering the high sequence similarity among alleles of different loci, which can be over 98% [15]. With regard to Mal d 1.04, two of the sequenced cultivars showed variant compositions for *Mal d 1.04 *that did not exactly fit with the relative SPT data found. Prima has the functional allele of *Mal d 1.04 *in duplex but has an intermediate allergenicity, whereas Fuji is the only intermediate-allergenic cultivar that had two pseudo-alleles for *Mal d 1.04 *(Table 2). This might indicate that *Mal d 1.04 *does not show dosage allele effects and cannot explain allergenicity without considering other factors. Alternatively, these two exceptions infer variant 03 of Mal d 1.06A to be more allergenic than variant 01, in which case the stronger effect of 03 in Fuji is counteracted by the absence of a functional allele for Mal d 1.04. Similarly, the milder effect of variant 01 of Prima is then compensated by the double dosage of a functional Mal d 1.04 allele. A stronger effect of variant 03 fits with the tendency of higher responses of the three heterozygous 01/03 cultivars compared to the two 01 homozygous cultivars.

The above described associations could be found due to the presence of allelic variation among the examined apple cultivars and due to performing a complexity reduction of the human variation by only analysing patients with mild SPT responses, thus reducing the effect of variation among humans for sensitivity to different allergens. Studies with larger patient sizes may probably benefit from further grouping to also account for genetically determined human variation in sensitivity to different (iso) allergen variants. Such grouping has probably to be based on allergy responses as no knowledge exists on the involved human genes neither on their allelic composition.

The finding that allergenicity depends on the presence and amount of some specific Mal d 1 isoforms is highly relevant for diagnostics tests and immunotherapy, and justifies additional research on a larger number of apple cultivars as well as atopic individuals. Since the first *Mal d 2 *and *Mal d 4 *genes have also been recently mapped [22] and the mapping of additional genes of these allergens is in progress, it will become possible to also investigate the effects of allelic composition of these Mal d allergens on the allergenicity of cultivars by association studies.

### Location of amino acid polymorphism in a 3D structure model

For *Mal d 1.06A*, high-allergenic cultivars have two putative genotypes, homozygous variant 01 or heterozygous variant 01 together with variant 03, whereas low-allergenic cultivars are homozygous for variant 02. The intermediate-allergenic cultivars contained the low allergenic variant 02 in combination with one of the high allergenic variants 01 or 03.

The three Mal d 1.06A variants differ at two amino acids: 13 V/I and 135V/A. Considering the three dimensional structure model of Mal d 1 [23], the first polymorphism is located in the first loop between the β1-strand and the α1-helix, the second is located in the α3-helix structure motif. The amino acid changes are all between hydrophobic amino acids but they have different side chains that may have an effect on the 3D structure of the protein and thus on epitope conformation.

### Expression of *Mal d 1 *genes in fruit

For specific *Mal d 1 *genes to be involved in allergenicity, expression in apple fruit is a prerequisite. Until now, mRNA expression for five genes was observed in mature fruit through both rtPCR [16,24] and EST sequences (unpublished), representing two genes (*Mal d 1.01 *and *-03E*) on LG 13 and three genes (*Mal d 1.02*, *-06A *and *-06B*) on LG 16., whereas mRNA based EST sequences of *Mal d 1.04 *were only found in mature leaves. Because the number of rtPCR studies on and EST sequences derived from fruit is still limited and usually only assessed at a single time moment, definitive mRNA based conclusions regarding *Mal d 1.04 *expression in fruit can not yet be made. Recently obtained proof for its expression in the epidermis of fruit [24] is not conclusive as the primers used can probably also amplify mRNA of *Mal d 1.05 *and *Mal d 1.06B*. From ESTs identified in cDNA libraries of ripe Gala fruit, tissue specific expression patterns were found. Mal d 1.01 and Mal d 1.02 ESTs were found in both skin and cortex, while Mal d 1.06A, 1.06B and 1.03E ESTs were only found in the skin of apple fruit. At this moment, there is evidence for the presence of two Mal d 1 proteins in apple fruit [17,18], the majority of Mal d 1 protein is Mal d 1.02 (Mal d 1b) and a minor part is Mal d 1.06A [15]. Interestingly, both genes are located on linkage group 16 where also *Mal d 1.04 *is located. These mRNA and protein data thus allow Mal d 1.06A to be involved in differences in allergenicity among cultivars. The current lack of support for the presence of Mal d 1.04 in fruit might indicate that the observed association is coincidental, but may as well be due to lack of extensive expression studies.

### Genotyping for *Mal d 1 *haplotypes

The *Mal d 1 *genes in LG 16 are tightly linked to each other [15]. This tight linkage can simplify the genotyping of additional cultivars, at least if their pedigree and the linkage phase of their parental alleles are known. In these cases, genotyping can be performed by a single representative, multi-allelic marker such as the Mal d 1.06A SSR marker. As linkage phases of the *Mal d 1 *genes of LG 16 are known for all 10 cultivars of our reference set but Discovery (Table 2), this simple and efficient approach was performed in this study for certain cultivars (Figure. 1).

## Conclusion

We have shown that differences in allergenicity among apple cultivars are associated with the allelic composition of two specific genes, *Mal d 1.04 *and *Mal d 1.06A*, which are both located on linkage 16 of *Malus domestica*. Furthermore, allele dosage effects are found relevant for *Mal d 1.06A*. Our findings indicate the need to reconsider the relevance of merely assessing total amounts of Mal d 1 protein in allergy research and diagnostic tests and warrant further research on the association of specific *Mal d 1 *isoforms and allergenicity among a larger group of cultivars and allergy sufferers.

## Methods

### Cultivars for cloning and sequencing

Eight cultivars were used for cloning and sequencing of *Mal d 1 *isoallergen genes: Golden Delicious (GD), Priscilla (PS), Ingrid Marie (IM), Cox (CO), Jonathan (JO), Red Delicious (RD), Fuji (FJ) and Discovery (DS). Including the two parental cultivars Prima (PM) and Fiesta (FS) used in our previous mapping study [15], a total of ten cultivars were under investigation. They were chosen for three reasons: (1) GD, JO, CO and RD are founders in many breeding programmes world wide, and RD, GD and FJ are main cultivars for apple production in the world; (2) GD, IM and PS are members in the pedigree from GD (hyper-allergenic) to Santana (ST) = [PS × Elstar (= GD × IM)] a hypo-allergenic cultivar; (3) JO, DS, PM and FS are the parents of three mapping progenies.

### Genomic cloning and sequencing of intron-containing Mal d 1 genes

For each cultivar, all seven intron-containing *Mal d 1 *genes were cloned and sequenced: *Mal d 1.05 *of linkage group (LG) 6; *Mal d 1.01 *of LG 13; and *Mal d 1.02*, *-1.04*, *-1.06A*, *-1.06B*, *-1.06C *of LG 16. Six primer pairs were used for cloning [15], of which those for *Mal d 1.04 *and *Mal d 1.05 *were newly designed (Table 6). The PCR amplification, cloning and sequencing procedures were described previously [15,22]. For all 10 cultivars, eight to ten clones for each gene were sequenced in both directions. Next, sequences were aligned and putative Single Nucleotide Polymorphisms (SNPs) were identified using the Seqman program (DNAstar, Madison, WI). The coding sequences were deduced and translated into amino acid sequences for alignments and assessment of protein variant with the GeneDoc program . New protein variants or gDNA alleles were named according to Gao et al. [15,22], following a modification of the allergen nomenclature guidelines [25].

**Table 6:** Cloning primers and PCR conditions.

**Gene**	**Primers^a^**	**Pfu^b ^Tm/cycles**	**Taq Tm/cycles**	**Reference sequence**
*Mal d 1.01*	F: ATCTCCAACACAATACTCTCAAC R: AAAGCCACACAACCTTCGAC	58/25	60/2	AY789236
*Mal d 1.02*	F: CATCCTTGGTAGTTGCTTTC R: ACCATAGAAACATATTAATTTAGT	52/25	54/2	AY789239
*Mal d 1.04*	F: CGTAGTTGGACAAGTGTCTTAGT R: AGGGTAACACACAAATTACATG	58/30	60/2	AY789242
*Mal d 1.05*	F: AGTTCATCATGGGTGTTTTC R: GGTAACACACAAATTACAAATATGC	53/30	55/2	AY789245
*Mal d 1.06A-C*	F: CATGGGTGTCCTCACATACGAAAC R: TTAGTTGTAGGCATCAGGATTG	55/25	57/2	AY789248
*Mal d 1.06C*	F: ATGGGTGTCCTCACATACGAAACT R: TTAGTTGTAGGCATCAGGATTGGCCACAAGGTG	62/30	64/2	AY789255

^a^Primers for *Mal d 1.04 *and *Mal d 1.05 *are new, others have been adopted from Gao et al. [15].

^b ^The PCR was performed in two steps starting with Pfu polymerase and finishing with super Taq [15,22].

### Cultivars for association studies

Allergenicity data were available for 6 out of the 10 cultivars for which we assessed allelic diversity [9]. Besides these six cultivars, eight additional cultivars were included in the association study. For these eight cultivars allergenicity data were available [9] and their allelic constitutions of the intron-containing *Mal d 1 *genes could be assessed by their pedigree relationships to the set of 10 sequenced cultivars.

For this, sequence-specific SNAP markers [23,27] were developed (Table 7) and applied to these cultivars. Primer design and test procedures were described previously [15,27]. Primers for the Mal d 1.06A SSR marker [15] were redesigned according to new sequence data obtained. The PCR reaction mixture for this SSR marker consisted of 2 μl 10× buffer, 1.2 μl MgCl_2 _(25 mM), 0.4 μl dNTPs (10 mM), 1μl of each primer (2 μM), 0.06 μl Taq polymerase (5 U/μl) and 1μl DNA (10 ng/μl) in a total volume of 20 μl. After an initial denaturation at 94°C for 2.5 min, the amplification was carried out for 34 cycles at 94°C for 30 s, 60°C for 30 s and 72°C for 1 min, and a final extension at 72°C for 5 min. PCR products were analysed on an ABI 377 (Applied Biosystems, Foster City, Calif.).

**Table 7:** Description of nine sequence-specific SNAP markers and one SSR marker used for the genotyping of cultivars (in bold: SNP, underlined are deliberate introduced SNP to increase specificity).

**Marker name^a^**	**Primers 5'-3'**	**SNPs detected^b^**	**Product (nt)**	**Tm^c^**	**Cycles^d^**
Mal d 1.010502	F: TGAAGCACAGGATTGAC**G**C**A** R: CCACACAACCTTCGACT**C**A	390A	346	56	35
Mal d 1.010501	F: AGCTGAAATCCTTGAA**C**GAA R: CAATGTTTCCCTTGGTG**A**G**A**	- 528T	426	55/53	10/30
Mal d 1.010503	F: TGAAGCACAGGATTGAC**A**C**G** R: CAATGTTTCCCTTGGTGT**C****G**	390G 528C	177	57/55	10/30
Mal d 1.0201-1	F: TCACTTTTGGTGAAGGTC**T**G R: AGGCGTATGAGTAGTTT**A**C**C**	- 402G	252	57	35
Mal d 1.0201-4	F: ATTATTTAGATGGTTTCGCT**A****T** R: TATGCGTCGGGGTGTCCCT**A****G**	227T 624C	439	55	35
Mal d 1.0201-3	F: GCCCTGGAACCATCAA**G****T**A**G** R: GGCTGCCTGTGAGACAA**C**T**G**	165G, 168G 342C	213	61	35
Mal d 1.04-6/7	F: CATCCCGAAGATTGCTCC**G****T** R: CCTTAGCATGGTAGTGGC**G**A**G**	109T 464C	395	57	35
Mal d 1.04-4	F: CAAGGAAGAGCATGTTAA**A**G**T** R: AGGGTAACACACAAATTACATG	516A, 518T	136	60	35
Mal d 1.06C0201-2	F: CCACCATTTTCTCCATTAACTT**C**A R: GCCTTAACATGCTCTTCCTTGATT	221A	360	62/60	5/35
Mal d 1.06A-SSR^**e**^	F: GGTGAAGGTTAGTTTAATTTCCACA R: ***GT***TTCACATAGCTGTATTCACTCCCT^f^	(CA) SSR		60	30

^a ^Names are according to Table 1;

^b^Position refers to the genomic sequence counted from the ATG starting codon;

^c,d ^PCR annealing temperature (Tm) and number of cycles, respectively. In case of two values, a touch-down PCR was performed in two steps.

^**e **^Note that although the SSR marker for *Mal d 1.06A *is gene specific, its is not sequence specific. In cases where a marker allele could be derived from both parents, linked markers have been used to assess the inheritance of protein variants to cultivars.

^f ^Nucleotides that are italicized were added to the 5' end of the initial primer to obtain the sequence GTTT, which serves a pig-tail [31].

Using the SNAP makers, the pedigree structures allowed us to follow the flow of the *Mal d 1 *alleles over generations by applying the Identity by Descent principle in the genotyping of cultivars [19]. In total 14 cultivars were thus available for association of protein variant composition with SPT responses.

### Allergenicity data

In this study, Skin Prick Test (SPT) responses were used to evaluate allergenicity. The SPT procedure and the history of the patients have been described previously [9]. In short, patients were recruited from the outpatient clinic of the department of Dermatology/Allergology of the UMCU. They all had birch pollinosis manifesting with rhinoconjunctivitis during the birch pollen season (April and May), as well as a positive SPT to fresh apple of at least half the diameter of the positive histamine control. All patients had a typical history of apple allergy, with oral allergy syndrome (OAS) symptoms like itching and mild swelling of the mouth, throat and sometimes rhinoconjuctivitis after eating an apple. SPT were performed on the flexor surface of the forearm using the prick-to-prick-technique according to Dreborg [28,29].

Histamine dihydrochloride (10 mg/ml) was used as a positive control, and the glycerol diluents of the SPT-extracts were used as negative control (ALK-ABELLO, Nieuwegein, The Netherlands). The wheal reaction (a small, itching elevation of the skin, as from the bite of an insect) was marked and transferred with transparent adhesive tape to a record sheet. The skin wheal areas were measured by computer scanning [30]. SPT responses for each cultivar were standardized by dividing the original wheal area of the prick by that obtained from the reference cultivar for high allergenicity Golden Delicious and multiplied by 100. Data have been derived from four experiments, three of which had been published previously [9]. For each experiment we used only a fraction of the data, this is only the data of patients with mild symptoms as preliminary experiments indicated that Mal d 1 is the major allergen to these patients, while other Mal d proteins seem to be major allergens to patients displaying more severe symptoms (Van de Weg, unpublished). Consequently, only 25%–50% of the patients of the previous experiments [9] and only 50% of the patients (4 out of 8) of the forth, new experiment were included. Finally, 11 different patients were involved. In order to combine data from these different experiments, responses of cultivars were expressed as a percentage of the response against the reference cultivar Golden Delicious. The final ranking results were obtained by averaging the responses from four different experiments. This study was reviewed and approved by the Ethics Committee of the University Medical Center Utrecht under document number 01–050. All patients provided written informed consent before enrolment in the study.

## Authors' contributions

LJWJG and KHS initiated this study. LJWJG, KHS, EVW and ZSG designed the experiment. ZSG, CIM and PA performed the gene cloning and sequences analysis. STHPB and ACK conducted the skin prick experiments. YHL tested and analysed the SSR markers. EVW performed the association analyses. ZSG, EVW, PA and LJWJG drafted the paper.
